# Analysis of commercially available snake antivenoms reveals high contents of endotoxins in some products

**DOI:** 10.1016/j.toxcx.2024.100187

**Published:** 2024-02-15

**Authors:** Gabriela Solano, Stuart Ainsworth, Adriana Sánchez, Mauren Villalta, Paola Sánchez, Gina Durán, José María Gutiérrez, Guillermo León

**Affiliations:** a Instituto Clodomiro Picado, Facultad de Microbiología, Universidad de Costa Rica, San José, Costa Rica; b Department of Infection Biology and Microbiomes, Institute of Infection, Veterinary and Ecological Sciences, University of Liverpool, Liverpool, L3 5RF, UK

**Keywords:** Endotoxin, *Limulus* amebocyte lysate test, Snake antivenom, Pyrogen

## Abstract

As injectable therapeutics, snake antivenoms must meet specifications for endotoxin content. The *Limulus* amebocyte lysate (LAL) test was used to evaluate the endotoxin content in several commercially available antivenoms released for clinical use. It was found that some products have endotoxin concentrations higher than the accepted limit for these contaminants. These results emphasize the need to include endotoxin determination as part of the routine evaluation of antivenoms by manufacturers and regulatory agencies.

## Highlights

• The *Limulus* amebocyte lysate test was used to evaluate the endotoxin content in commercially available antivenoms.
• Some tested antivenoms contain endotoxin concentrations higher than the accepted limit for these contaminants.
• Testing for endotoxin contamination of antivenoms must be routinely performed by manufacturers and regulatory agencies.

Snakebite envenomation is an important public health problem that primarily affects rural communities in tropical and subtropical countries (World Health Organization, 2017). The only scientifically validated medication for the treatment of these medical emergencies is the parenteral administration of antivenoms (i.e., formulations of whole immunoglobulins, or their F (ab')_2_ or Fab fragments, purified from plasma of animals immunized with snake venoms; (World Health Organization, 2017).

Antivenoms may induce early adverse reactions, whose frequency varies depending on the products (León et al., 2013). Many of these reactions are related to the foreign nature of equine immunoglobulins, as well as to the presence of protein aggregates, being also related to the total antivenom protein administered. In addition, early reactions could be due to the contamination of the antivenom with endotoxins (León et al., 2013). Adverse reactions produced by endotoxins (i.e., pyrogenic reactions) are clinically evidenced as fever, myalgia, headache, nausea, chills, increased heart rate, hypotension, renal failure, circulatory collapse, respiratory distress syndrome. Severe reactions have uncommonly caused fatalities (Skarnes et al., 1981; Williams, 2007).

Endotoxins are macromolecular complexes of lipopolysaccharides (LPS), proteins, and phospholipids released from the outer membrane of Gram-negative bacteria during microbial growth (Jackie et al., 2019). The production of batches of antivenom contaminated with endotoxins must be prevented by the implementation of current good manufacturing practices (cGMPs). Several techniques can be used to reduce the in-process contamination with endotoxins during hyperimmune plasma fractionation (Sheraba et al., 2023). However, these procedures should not be used in substitution of cGMPs, to avoid contamination problems upstream in the manufacturing process.

The antivenom production process involves several stages where the in-process products might be exposed to microbial contamination. To reduce the likelihood of this risk, all the facilities, equipment and procedures used during manufacture, from horse venipuncture and plasma harvesting to product vial marketing, must be properly designed and validated. Manufacturing standards and performance must be continuously monitored by following cGMPs. As antivenoms are formulated with preservatives and are subjected to membrane filtration, these products are generally sterile.

However, whilst these processes ensure the sterility of the antivenom, they are unable to remove bacterial endotoxins generated by microbial contamination during the manufacturing process. When they reach their critical micellar concentration, endotoxins form micelles with variable size, depending on their extent of aggregation. The difference in the molecular weight of endotoxin micelles and antivenom antibodies is not large enough to separate these molecules by ultrafiltration (10–30 kDa MWCO) or microfiltration (0.2 μm cutoff). Consequently, the endotoxins present in the antivenoms prior to sterilization are not removed during the sterilizing filtration, and they may end up in the final product, representing a serious concern in antivenom manufacture owing to the toxicity of these microbial contaminants, including pyrogenicity and other deleterious effects.

To prevent the manufacture of contaminated antivenoms, the endotoxins content in these products must be rigorously controlled by manufacturing laboratories and regulatory entities in the country where the antivenom will be used. The presence of endotoxins can be assessed by the *in vivo* rabbit pyrogen test, or by several *in vitro* assays (World Health Organization, 2017). The *Limulus* Amebocyte Lysate (LAL) assay is one such *in vitro* technique widely used in the pharmaceutical industry for the detection and quantification of endotoxins (Solano et al., 2015; Tamura et al., 2021). In this work, we used the LAL test to assess the endotoxin content of several commercially available antivenoms distributed in different regions of the world.

The following antivenoms were used: Antivenom-2 Polyvalent Anti-Snake Venom Sera (Batch: SAS42202; Exp. Date: Oct/2023; Scientific Studies & Research Centre, Syria); Biosave Polyvalent anti-snake venom serum (Batch: 4700223; Exp. Date: Apr/2025; PT Biofarma, Indonesia); Bothrofav (Batch: P4A561V; Exp. Date: Oct/2020; Micropharm, United Kingdom); Faboterápico Polivalente Antiviperino (Batch: SV189; Exp. Date: Aug/2015; Birmex, México); Hemato Polyvalent Snake Antivenin (Batch HP00415; Exp. Date: Sep/2020; Queen Saovabha Memorial Institute, Thailand); Neuro Polyvalent Snake Antivenin (Batch HP00515; Exp. Date: Sep/2020; Queen Saovabha Memorial Institute, Thailand); NORAF-Premium Combipack of Snake Venom Antiserum for North Africa (Batch NORAF-001; Exp. Date: Dec/2021; Premium Serums and Vaccines PVT. LTD, India); Panafrican EchiTAb-plus ICP (Batch: 7030723PALF; Exp. Date: Jul/2028; Instituto Clodomiro Picado, Costa Rica); Polyvalent Snake Antivenom (Batch: PSn73H; Exp. Date: Jun/2012; Antivenom Experts in Saudi National Guard, Saudi Arabia); SAIMR Polyvalent Antivenom (Batch: BB01446; Exp. Date: Jul/2015; South African Vaccine Producers LTD, South Africa); Snake Venom Antiserum (Batch: A5309094; Exp. Date: Sep/2013; Bharat Serums and Vaccines Limited, India); Snake Venom Antiserum African-10 (Batch: 07AS16001; Exp. Date: Dec/2019; Vins Bioproducts Limited, India); Snake Venom Antiserum I.P. (Batch: 0183/10–11; Exp. Date: Nov/2014; Vins Bioproducts Limited, India); Soro Antibotrópico (Batch: 06 09168/C; Exp. Date: Aug/2009; Instituto Butantan, Brazil); Soro Antibotropico FUNED (Batch: 051 018–33; Exp. Date: Nov/2008; Fundação Ezequiel Dias, Brazil); Suero antibotrópico Polivalente (Batch: 01200407; Exp. Date: Dec/2010; Instituto Nacional de Salud de Perú); Suero Antiofídico Centroamericano Biol-CLB (Batch: 6365; Exp. Date: May/2023; Instituto Biológico Argentino SAIC, Argentina); Suero Antiofídico Polivalente (Batch: 158; Exp. Date: Feb/2013; Centro de Biotecnología, Facultad de Farmacia, Universidad Central de Venezuela, Venezuela); Suero Antiofídico Polivalente Biol (Batch: 1122; Exp. Date: Jun/2012; Instituto Biológico Argentino SAIC, Argentina).

The LAL assay was performed in duplicate, using the gel clot method. Specifically, 0.2 mL of several dilutions of antivenoms in LAL reagent water (ACC, cat # WP1001) were added to a single test vial of LAL reagent (Pyrotell®; Associates of Cape Cod Incorporated, cat # 65003, East Falmouth, MA, USA), which sensitivity (1λ) is 0.03 EU/mL. Afterwards, the vials were incubated at 37 ± 1 °C for 60 ± 2 min. Then, the vials were gently inverted 180° to determine gelation of the mixture (United States Pharmacopeia, 2023). Inhibition controls were prepared by spiking the antivenom dilutions with the amount of LPS standard (Associates of Cape Cod Incorporated, ACC cat #E0005) that corresponds to twice the sensitivity of the LAL reagent (2λ). Positive controls were prepared with LPS (2λ) and LAL reagent water, while negative controls were prepared with the LAL reagent water used to prepare the dilutions. The endotoxin content of each antivenom was compared to its corresponding endotoxin limit, which was calculated as the ratio K/M, where K is the threshold pyrogenic dose of endotoxin per kilogram of body weight (5 endotoxin units (EU)/kg), and M is the maximum total dose of antivenom administered to a 70 kg-patient in 1 h (United States Pharmacopeia, 2023). The maximum total dose corresponds to the dose recommended by the manufacturer for the treatment of severe envenomations, as indicated in the product inserts.

We tested 19 batches of antivenoms manufactured in several countries which are commercially available. To ensure the anonymity of the antivenoms analyzed, a coding system was used, which does not correspond to the order in which antivenoms were described above. The majority of the antivenoms tested (A to N, Table 1) met the endotoxin specification, even in the case of products that were expired. On the other hand, antivenoms O to S show contents of endotoxins higher than the accepted limit (Table 1), even though all the products analyzed were released and distributed for the treatment of snakebite envenomations. Endotoxins have been identified as the etiological agent of pyrogenic reactions (Martich et al., 1993). Therefore, the clinical use of antivenoms having endotoxin contents higher than the accepted values is likely to result in the induction of pyrogenic reactions, which can worsen the clinical prognosis of the cases.

**Table 1:** Content of endotoxins in commercially available snake antivenoms.

Antivenom	Dilution factor												Endotoxin content (EU/mL)	Endotoxin limit (EU/mL)
	25	50	70	100	200	400	580	800	1600	3200	6400	12800		
A	–	–		–	–	–							<0.75	8.75
B	–	–		–	–	–							<0.75	8.75
C	–	–		–									<0.75	17.50
D		–		–									<1.50	2.33
E		–		–									<1.50	1.75
F		–		–	–	–							<1.50	3.50
G		–											<1.50	35.00
H		–		–									<1.50	2.33
I		–		–									<1.50	3.50
J		++	–	–									<2.10	2.33
K		++		–	–								<3.00	3.50
L				–	–								<3.00	17.5
M				++	–	–		–	–	–			<4.20	4.38
N						++	–	–	–				<17.40	17.50
O				++	++	++		++	++	–	–		≥48.00	35.00
P				++	++	++		++	++	–			≥48.00	3.50
Q								++	++	++	–		≥60.00	3.50
R					++	++		++	++	++	–	–	≥96.00	17.50
S					++	++		++	++	++	++	–	≥192.00	17.50

*The endotoxin limit was calculated as the ratio K/M, where K is the threshold pyrogenic dose of endotoxin per kilogram of body weight (5 EU/kg), and M is the maximum total dose administered to a 70 kg-patient for 1 h (United States Pharmacopeia, 2023). The maximum total dose corresponds to the dose recommended by the manufacturer for the treatment of severe envenomations, as indicated in the prospects of the products. All inhibition controls showed a positive result, ensuring the tests validity.

The main limitation of this study is that several antivenoms were tested after their expiry date. The broad differences in expiration (from Nov/2008 to Jul/2028) are a source of heterogeneity in the products being evaluated. However, once sealed, antivenom vials or ampoules are protected from external contamination. Therefore, if the seals are not broken and the glass remains intact, the content of endotoxins in the products, provided they are sterile, should not increase during shelf-life and after the expiry date. This was evident in our results, since the content of endotoxins in the products evaluated was not related to whether the batches used were expired or not.

Our findings underscore the need to evaluate the endotoxin content of antivenoms by manufacturers and regulatory agencies, as well as to include this issue in Pharmacopeias and guidelines for antivenom production and quality control. Moreover, our data stress the need to incorporate specifications for maximum allowable endotoxin content of antivenoms in the Target Product Profiles (TPP) for animal-derived antivenoms for sub-Saharan Africa, recently issued by the World Health Organization (World Health Organization, 2023), and in the new TPPs being prepared.

## Ethical statement

This manuscript presents an experimental study performed following the standard procedure of scientific ethics.

## CRediT authorship contribution statement

**Gabriela Solano:** Writing – original draft, Methodology, Investigation. **Stuart Ainsworth:** Writing – original draft, Funding acquisition, Conceptualization. **Adriana Sánchez:** Writing – review & editing, Validation, Resources. **Mauren Villalta:** Writing – review & editing, Supervision, Resources. **Paola Sánchez:** Writing – review & editing, Validation, Resources. **Gina Durán:** Writing – review & editing, Validation, Resources. **José María Gutiérrez:** Writing – original draft, Project administration, Funding acquisition. **Guillermo León:** Writing – original draft, Project administration, Conceptualization.

## Declaration of competing interest

The authors declare the following financial interests/personal relationships which may be considered as potential competing interests: Gabriela Solano, Adriana Sanchez, Mauren Villalta, Paola Sanchez, Gina Duran, Jose Maria Gutierrez and Guillrmo Leon work at Instituto Clodomiro Picado, where one of the antivenoms tested in this study is manufactured.

Guillermo Leon Montero reports financial support was provided by 10.13039/100010269Wellcome Trust. Stuart Ainsworth reports financial support was provided by United Kingdom Research and Innovation.

## Data Availability

The data is available upon request"
